# Sixteen-and-a-half syndrome with metastatic pons tumor: A case report

**DOI:** 10.1097/MD.0000000000018006

**Published:** 2019-11-22

**Authors:** Shin-Myeong Choi, Tae Gi Kim, Junkyu Chung, Jin-Ho Joo, In-Ki Park, Sang Woong Moon, Jae-Ho Shin

**Affiliations:** a Department of Ophthalmology, Kyung Hee University Hospital at Gangdong, Kyung Hee University School of Medicine; b Division of Ophthalmology, Department of Medicine, Kyung Hee University Graduate School; c Department of Ophthalmology, Kyung Hee University Hospital, Kyung Hee University School of Medicine, Seoul, Korea.

**Keywords:** nonsmall cell lung cancer, ophthalmoplegia, 16-and-a-half syndrome

## Abstract

**Rationale:**

One-and-a-half syndrome (OAAH) is characterized as the combination of ipsilateral horizontal gaze palsy and internuclear ophthalmoplegia. OAAH syndrome accompanied with 7th and 8th cranial nerve palsy is called 16-and-a-half syndrome. We aimed to report the case of 16-and-a-half syndrome with metastatic pons tumor.

**Patient concerns:**

A 57-year-old male diagnosed with nonsmall-cell lung cancer (NSCLC) with brain metastasis occurring 15 months ago was referred to our clinic with the chief complaint of horizontal diplopia and right gaze palsy.

**Diagnosis:**

According to the patient symptom, ocular examination, and radiographic findings, he was diagnosed as 16-and-a-half syndrome which was caused by brain tumor metastasis from NSCLC.

**Interventions:**

We referred him to hemato-oncology department and he was treated with radiation and supportive therapy.

**Outcomes:**

Unfortunately, the patient passed away 1 month later without improvement of ophthalmoplegia.

**Lessons:**

The clinical findings of our case indicate 16-and-a-half syndrome caused by brain tumor metastasis from NSCLC, which to our knowledge has not been previously reported. The case highlights a rare cause of OAAH spectrum disease and the importance of a systemic work-up including associated neurologic symptoms and brain imaging in patients with horizontal gaze palsy.

## 1 Introduction

One-and-a-half syndrome (OAAH) has symptoms of ipsilateral conjugate horizontal gaze palsy and ipsilateral internuclear ophthalmoplegia.^[1]^ It manifests as a lesion-sided eye with complete horizontal gaze palsy (one), while the other side eye can only abduct by half. Anatomically, OAAH occurs when the lesion invades the brain structures responsible for horizontal movement, which are the paramedian pontine reticular formation (PPRF), abducens nucleus, and medial longitudinal fasciculus (MLF).

This syndrome was 1st mentioned by Fisher in 1967^[2]^ and OAAH along with different cranial palsies introduced since then are categorized under the nomenclature of “X-and-a-half syndrome.” OAAH with facial nerve (VII) palsy is called “8-and-a-half syndrome” according to simple addition (1 + 7). OAAH in combination with facial (VII) and cochlear (VIII) nerve palsy was 1st described as “16-and-a-half syndrome” in a case of brainstem infarction in 2011 by Cummins et al.^[3]^ The most common cause of OAAH spectrum disease is brainstem cerebrovascular pathologies (infarction) or demyelinating causes (e.g., multiple sclerosis).^[1,4]^ Other rare causes include head trauma, tuberculoma, and metastatic brain tumors.^[5,6]^

At the clinic, we saw 57-year-old male patient with a chief complaint of diplopia and horizontal gaze impairment with facial palsy and neurosensory hearing loss. Radiologic findings suggested metastasis of lung cancer to the pons area. He was diagnosed with a 16-and-a-half syndrome tumor involving PPRF, MLF, and the 7th and 8th cranial nuclei. To the best of our knowledge, 16-and-a-half syndrome caused by a metastatic brain tumor from lung cancer has not been reported, so we report this 1st case here with a review of the literature.

Patient has provided informed consent for publication of the case.

## 2 Case report

A 57-year-old male patient was referred to our clinic with the chief complaint of sudden onset diplopia in the left gaze and right gaze palsy. He had been diagnosed with stage IV nonsmall-cell lung cancer with hemisphere metastasis 15 months ago and was admitted to our hospital for chemotherapy.

**Figure 1:**
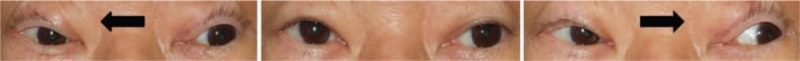
Right exotropia of 20 prism diopters in primary gaze. He had total right gaze palsy and limitation of adduction of the right eye in left gaze.

No history of ophthalmologic diagnosis or surgery was found in the past. During ocular examination, his best-corrected distance visual acuity was found to be 20/63 in the right eye and 20/200 in the left. His intraocular pressure, measured using a noncontact tonometer, was 18 mm Hg in both eyes. Pupillary light reflex was normal on both sides. In the primary gaze, he presented with 20-prism-diopter exotropia in the left eye. Extraocular movement test with 9 positions of gaze showed intact vertical eye movements but horizontal gaze palsy, while abduction of the left eye was also seen. Bilateral conjugate right gaze palsy and a right eye adduction deficit appeared in the left gaze. Additionally in the left gaze, only left eye abduction was possible, accompanied by abducting nystagmus (Fig. 1).

**Figure 2:**
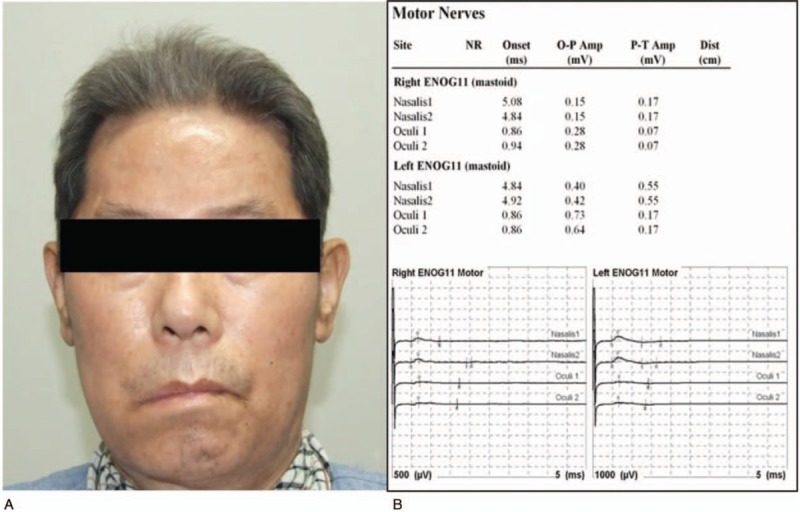
(A) He had right facial palsy with drooping of the right lower lips. (B) Electroneurography indicates right nasalis and oculi muscle excitation were lower than that in the left side by 30% (0.12/0.55) and 41% (0.07/0.17), respectively.

**Figure 3:**
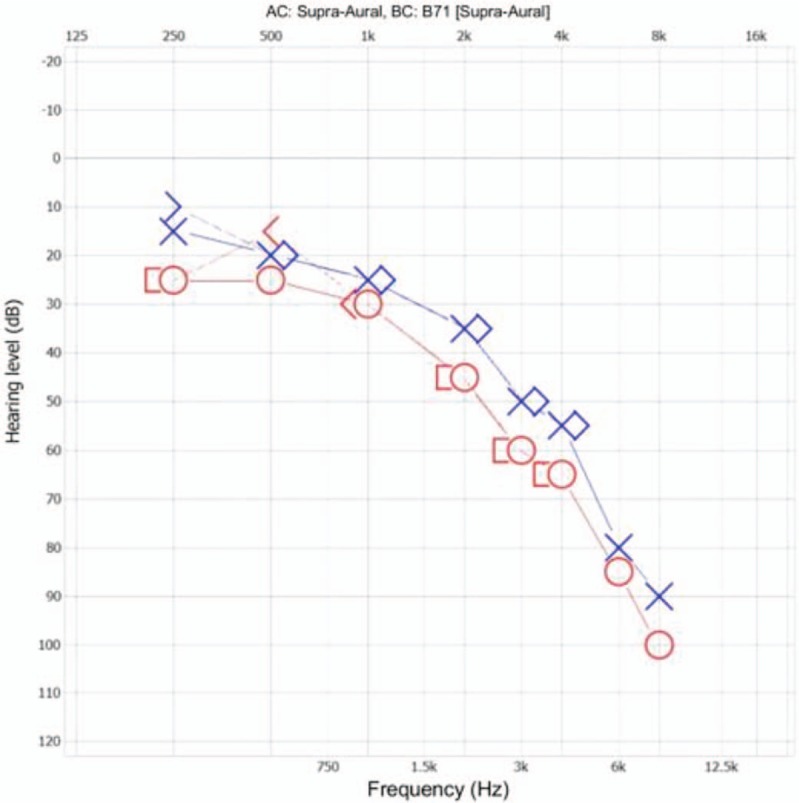
Pure tone audiometry presenting right-sided air conduction and bone conduction hearing level lower than on the left side, indicating sensorineural hearing loss of the right ear.

Neurologic examination revealed right-sided facial palsy with right upper eyelid ptosis, an inability to close the right eye, and drooping of the right corner of the mouth. Facial electromyography results indicated that right-sided nasalis and oculi muscle excitation were lower than on the left side by 30% and 41%, respectively (Fig. 2). He was diagnosed with House–Brackmann grade III right-sided facial palsy. He also complained of right-sided hearing loss from 6 months ago. Pure tone audiometry revealed that air and bone conduction levels of the right ear were lower than those of the left (Fig. 3). The Weber test was lateralized to the left ear and Rinne test showed air conduction greater than bone conduction in the right ear, suggesting sensorineural hearing loss in the right ear.

**Figure 4:**
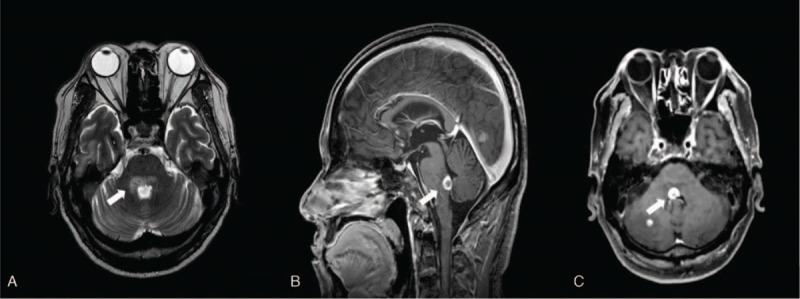
(A) Axial T2-weighted magnetic resonance imaging (MRI) image of the patient showing hyperintense lesion at the right tegmental pons (arrow). (B, C) Multiplanar reconstruction MRI saggital and axial images showing a high-signal-intensity lesion on the right pontine tegmentum (arrow).

Brain magnetic resonance imaging (MRI) revealed a hyperintense lesion involving the right pontine tegmentum (Fig. 4). The tumor had invaded the region of the PPRF, MLF, and 7th and 8th nuclei lateral to the 4th ventricle. This radiologic finding suggested newly diagnosed additional brain metastasis in the pons area.

He continued the chemotherapy and radiation therapy for the treatment of lung cancer and brain metastatic cancer. Despite treatment, the symptoms worsen and the patient was deceased after 4 months.

## 3 Discussion

The excitation signal that causes horizontal eye movement starts from the frontal eye field and is transmitted through the midbrain to the PPRF. The PPRF is located ventrally to the abducens nucleus and transmits an excitation signal to the ipsilateral abducens nucleus. The abducens nucleus receiving this signal stimulates the lateral rectus of the ipsilateral eye and simultaneously projects it upward through the contralateral MLF, which is located dorsally to the PPRF, to send signals to the oculomotor nucleus and medial rectus of the contralateral eye. This process makes conjugate eye movement possible by contraction of the lateral rectus of 1 eye and the medial rectus of the contralateral eye simultaneously. Any disruption involving the PPRF or abducens nucleus leads to lesion-sided horizontal gaze palsy. In the case of involvement of the ipsilateral MLF, the signal from the contralateral abducens nucleus will not be transmitted to the ipsilateral medial rectus muscle, resulting in an ipsilateral eye adduction deficit called internuclear ophthalmoplegia (INO).

A lesion involving both the PPRF or abducens nucleus and MLF manifests as horizontal gaze palsy (one) with contralateral adduction deficit (half). This was 1st reported and named by Miller Fisher in 1967^[2]^ as “1-and-a-half syndrome.” He reported 2 cases of OAAH caused by pons infarction, with both cases presenting with 1 eye fixed in the central position and the other eye capable of abducting.

As reported in our case, OAAH presents not only horizontal gaze palsy but also contralateral eye exotropia in primary gaze and abducting nystagmus, which are characteristic of INO. The exact mechanism is unknown to date, but Zee et al^[7]^ suggested this could be due to excessive development of neurofiber domination on the lateral rectus muscle to overcome the paralyzed medial rectus muscle.

Brain structures that regulate conjugate horizontal gaze such as the PPRF, abducens nucleus, and MLF are located on the tegmental pons. Additionally, the tegmental pons includes lower motor neurons of the facial nerve and cochlear nucleus complex near the PPRF and MLF. Therefore, the range of involvement of the lesion in the pons determines the neurologic symptoms associated with OAAH. In 1998, Eggenberger^[4]^ reported three patients with OAAH who presented with peripheral facial nerve palsy related to pons infarction. The combination of clinical findings (OAAH plus 7th cranial nerve) made him label it as “8-and-a-half syndrome.” Since then, several other subtype syndromes have been reported according to the neurologic symptoms associated with OAAH, with 16-and-a-half syndrome being 1 of them: specifically, Cummins et al^[3]^ identified unilateral hearing loss in a patient with 8-and-a-half syndrome due to cerebral infarction involving the 8th cranial nerve nucleus and 1st named it “16-and-a-half syndrome” in 2011.

**Figure 5:**
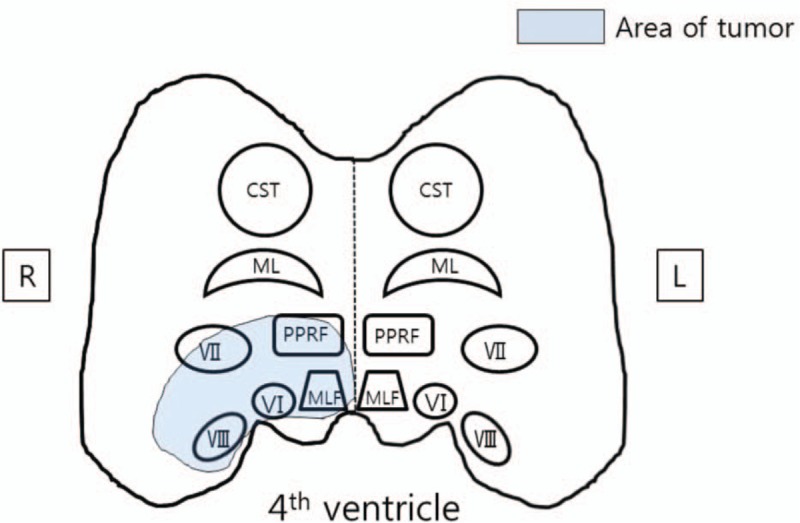
Schematic diagram of the pontine tegmentum. Area of the tumor (circle) in our patient involves the right MLF, abducens, and facial and vestibulocochlear nuclei. CST = corticospinal tract, ML = medial lemniscus, MLF = medial longitudinal fasciculus, PPRF = paramedian pontine reticular formation, VI = abducens nucleus, VII = facial nucleus, VIII = vestibulocochlear nucleus.

In the present case, we experienced a patient with OAAH with right sensory hearing deficiency and facial nerve palsy diagnosed with a new metastatic pons tumor found during MRI. The metastatic pons tumor had invaded the 6th, PPRF, MLF, and 7th and 8th cranial nerve nuclei, which is located lateral to the 4th ventricle (Fig. 5). This anatomical lesion was consistent with the clinical manifestation of 16-and-a-half syndrome.

The causes of OAAH spectrum disease include pons infarction and hemorrhage, demyelination such as multiple sclerosis, head trauma, and, rarely, metastatic tumors.^[5,8]^ OAAH caused by metastatic tumor from breast and renal cell cancer have been reported.^[6,9]^ In this case, we experienced an instance of 16-and-a-half syndrome caused by metastatic lung cancer with ipsilateral neurosensory hearing defect and facial palsy consistent with MRI findings.

To the best of our knowledge, 16-and-a-half syndrome caused by metastatic pons tumor from lung cancer has never been reported. Thus, this case represents another cause of the 16-and-a-half syndrome and emphasizes that a systemic neurologic workup including radiologic imaging is necessary in patients with eye movement impairment.

## Author contributions

**Conceptualization:** In-Ki Park.

**Formal analysis:** Jin-Ho Joo.

**Funding acquisition:** Junkyu Chung.

**Investigation:** Tae Gi Kim.

**Supervision:** Sang Woong Moon.

**Writing – original draft:** Shin-Myeong Choi.

**Writing – review & editing:** Jae-Ho Shin.
